# The Novel Acromial Angle is associated with the severity of full-thickness rotator cuff tear: a retrospective radiographic study

**DOI:** 10.3389/fmed.2026.1880699

**Published:** 2026-09-22

**Authors:** Zhipeng Hou, Jiabao Dong, Quqiao Wan, Wenzhao Shang, Kan Li, Hao Zhu

**Affiliations:** 1 Department of Orthopaedics, Affiliated Yueqing Hospital of Wenzhou Medical University, Wenzhou, China; 2 Department of Orthopaedics, Huzhou Central Hospital, Fifth School of Clinical Medicine of Zhejiang Chinese Medical University, Huzhou, China

**Keywords:** MRI, Novel Acromial Angle, rotator cuff tear, shoulder morphology, thickness

## Abstract

**Purpose:**

To investigate the relationship between the Novel Acromial Angle (NAA) and the severity of rotator cuff tears, including partial bursal-side and full-thickness tears.

**Methods:**

This retrospective cohort study included 386 patients who underwent both scapular outlet-view radiography and shoulder MRI at our hospital between September 2023 and April 2025. Based on MRI findings, patients were classified into a symptomatic MRI-negative control group, a partial bursal-side tear group, or a full-thickness tear group. The NAA was measured and compared among the groups.

**Results:**

The mean NAA was significantly smaller in both tear groups than in the symptomatic MRI-negative control group (*p* < 0.01). A smaller NAA was significantly associated with larger full-thickness tear size (*p* < 0.01). The area under the receiver operating characteristic curve (AUC) for identifying full-thickness tears was 0.76, which was higher than the AUC for partial tears (0.67). However, NAA was not significantly associated with the depth of partial bursal-side tears (*p* > 0.05).

**Conclusion:**

NAA was significantly associated with the presence and severity of full-thickness rotator cuff tears, with smaller angles corresponding to larger tears. NAA may be a useful radiographic parameter for preoperative planning and for evaluating the potential need for acromioplasty, particularly in patients with full-thickness tears.

## Introduction

Rotator cuff tears are among the most common causes of shoulder pain and are associated with both intrinsic and extrinsic factors (1). Among the extrinsic factors, acromial morphology is particularly important. Neer (2) proposed that most rotator cuff tears result from mechanical impingement between the rotator cuff and the acromion. Bigliani et al. (3) classified acromial morphology as flat, curved, or hooked, with hooked acromia being most strongly associated with rotator cuff tears. Accordingly, several morphologic parameters have been developed to investigate the relationship between scapular anatomy and rotator cuff tears (4–6), including the critical shoulder angle (CSA) and acromion index (AI) (7–9). The AI quantifies the lateral extension of the acromion, whereas the CSA incorporates both glenoid inclination and lateral acromial extension. Numerous studies have demonstrated associations between CSA, AI, and rotator cuff tears. More recently, investigators have examined their relationships with tear type and size, reporting larger CSA and AI values in patients with full-thickness tears (10).

However, both CSA and AI are measured on anteroposterior scapular radiographs. In clinical practice, scapular outlet-view radiographs are commonly used to assess acromial morphology and the potential need for acromioplasty during surgical planning. We therefore introduced the NAA and previously investigated its association with rotator cuff tears (11). Because the NAA can be measured directly on a clinically relevant scapular outlet view, the present study extends our previous findings by examining its association with tear severity, including massive tears. This study retrospectively evaluated the relationship between NAA and partial or full-thickness rotator cuff tears to provide evidence relevant to decisions regarding acromioplasty.

## Materials and methods

### Clinical data

This was a retrospective cohort study. A total of 386 complete imaging datasets were randomly and retrospectively selected from individuals who underwent scapular outlet view X-ray and shoulder MRI scans at our hospital between September 2023 to April 2025. Patients meeting the following criteria were included: (1) no infectious diseases of the shoulder joint; (2) no neoplastic diseases of the shoulder joint; (3) no traumatic diseases of the shoulder joint; (4) no congenital deformities of the shoulder joint; (5) no history of prior shoulder joint surgery. Among them, the symptomatic MRI-negative control group comprised 134 cases (78 males, 56 females; age: 59.1 ± 14.5 years). The partial bursal-side rotator cuff tear group comprised 126 cases (49 males, 77 females; age: 57.7 ± 9.5 years). The full-thickness rotator cuff tear group comprised 126 cases (35 males, 91 females; age: 62.2 ± 10.8 years).

Based on the presence and characteristics of rotator cuff tears on shoulder MRI (12), patients were divided into three groups: Group 1 (symptomatic MRI-negative control group), Group 2 (partial bursal-side rotator cuff tear group), and Group 3 (full-thickness rotator cuff tear group). Patients in the partial bursal-side tear group and the full-thickness tear group all underwent arthroscopic surgery, where the diagnosis was confirmed intraoperatively. The control group consisted of patients who had shoulder pain but no rotator cuff tear on MRI.

The size of full-thickness rotator cuff tears was classified according to Cofield (18) into four groups: small tear (<1 cm), medium tear (1–3 cm), large tear (3–5 cm), and massive tear (>5 cm). Partial bursal-side tears were graded based on tear depth into three groups: Grade I (1–3 mm), Grade II (3–6 mm), and Grade III (≥6 mm or > 50% of the full tendon thickness).

### Data measurement method

#### Measurement of Novel Acromial Angle

The measurement method for the Novel Acromial Angle (NAA) followed the technique described by Hou et al. (11) (Figure 1): On the scapular outlet view X-ray, the anterior and posterior edges of the undersurface of the acromion base were identified as points A and B, respectively. Point C was defined as the point located at the anterior one-third of line AB. A perpendicular line was drawn from point C to line AB, intersecting the undersurface of the acromion base at point D. Lines AD and BD were then drawn. Angle ADB is defined as the NAA.

**Figure 1:**
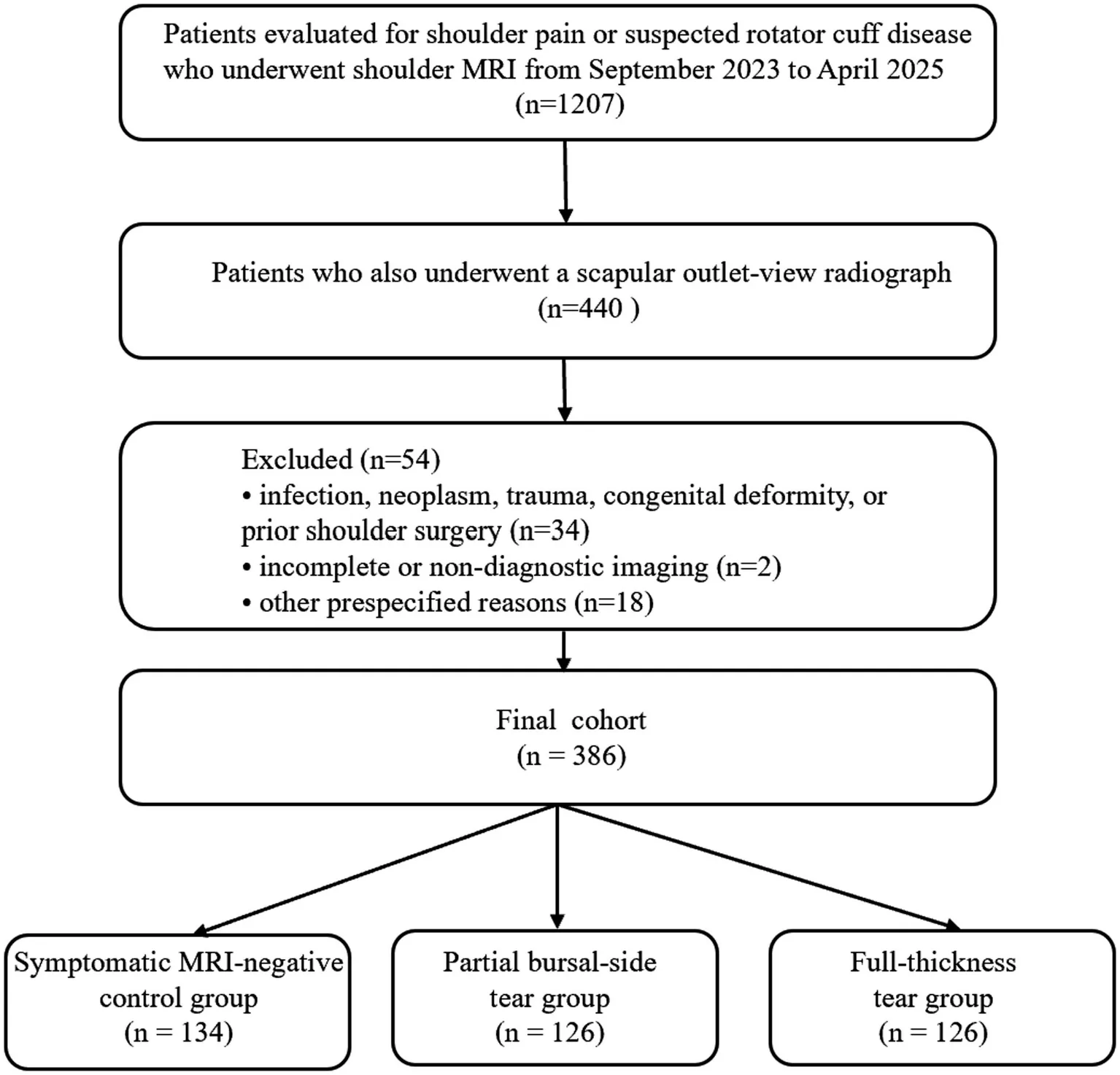
Patient selection flow diagram.

#### Measurement of full-thickness rotator cuff tear size

The coronal and axial planes of the shoulder MRI were synchronized. The tear was visualized on the coronal plane to identify its start and end points (Figure 2), and the measurement was then performed on the synchronized axial plane (Figure 3).

**Figure 2:**
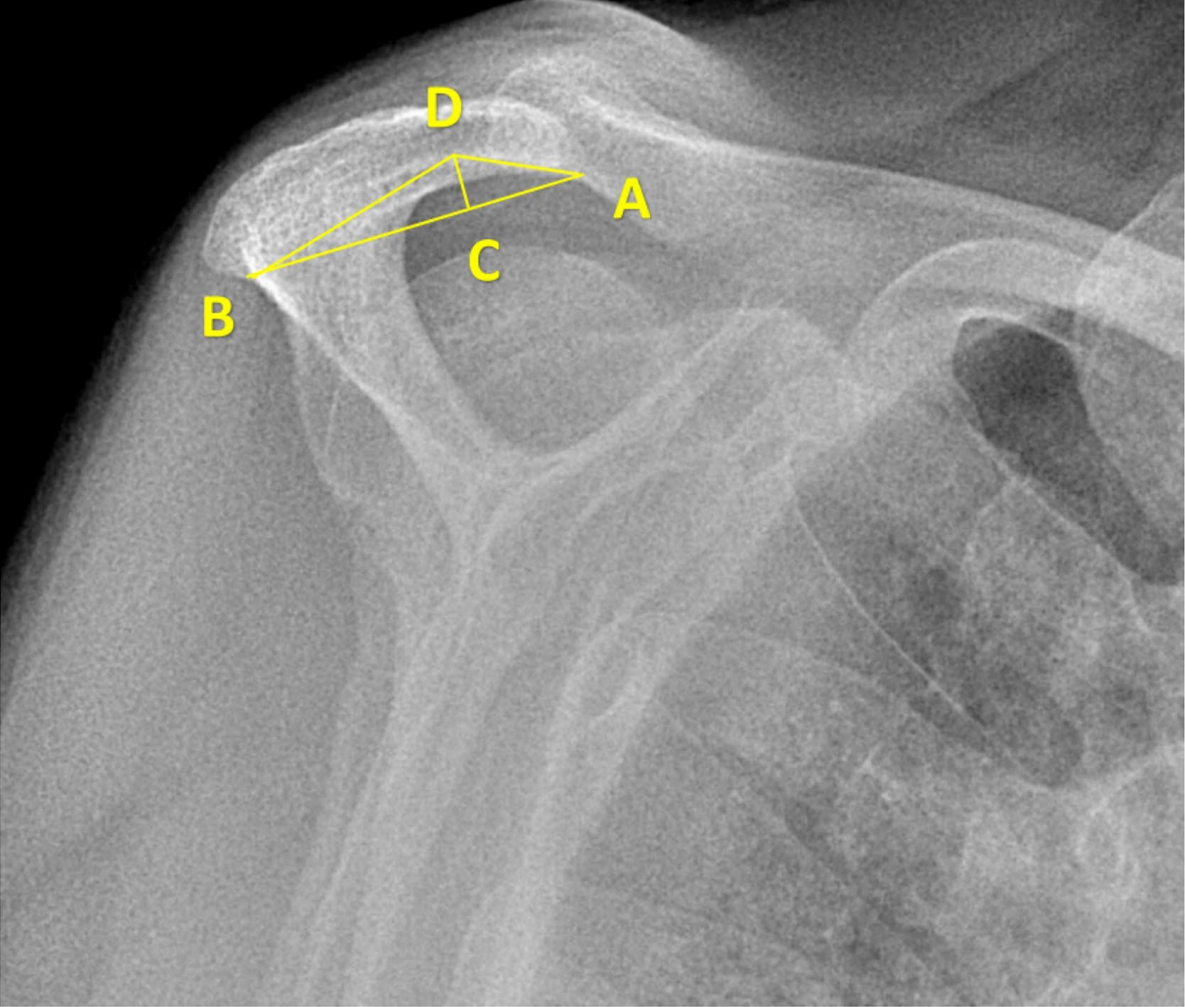
Measurement of NAA. A: anterior edge of the acromial base; B: posterior edge of the acromial base; C: the point at the anterior third of line AB; D: A point on the acromial base where the line connecting it to point C is perpendicular to line AB.

**Figure 3:**
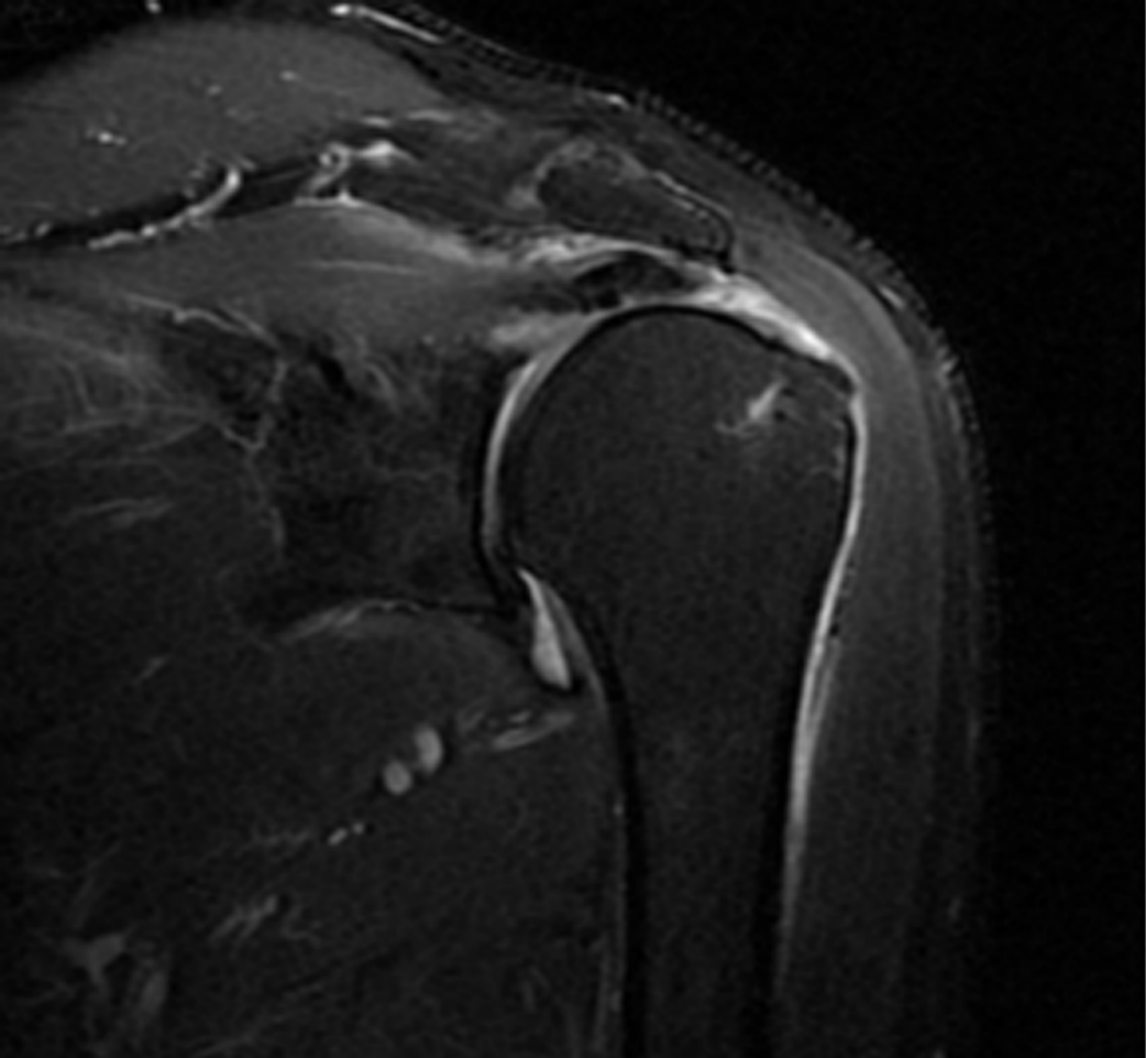
Coronal MR image of a full-thickness rotator cuff tear.

#### Measurement of partial bursal-side rotator cuff tear depth

On the coronal plane of the shoulder MRI, the maximum depth of the tear opening is taken as the depth of the partial bursal-side rotator cuff tear (Figure 4).

**Figure 4:**
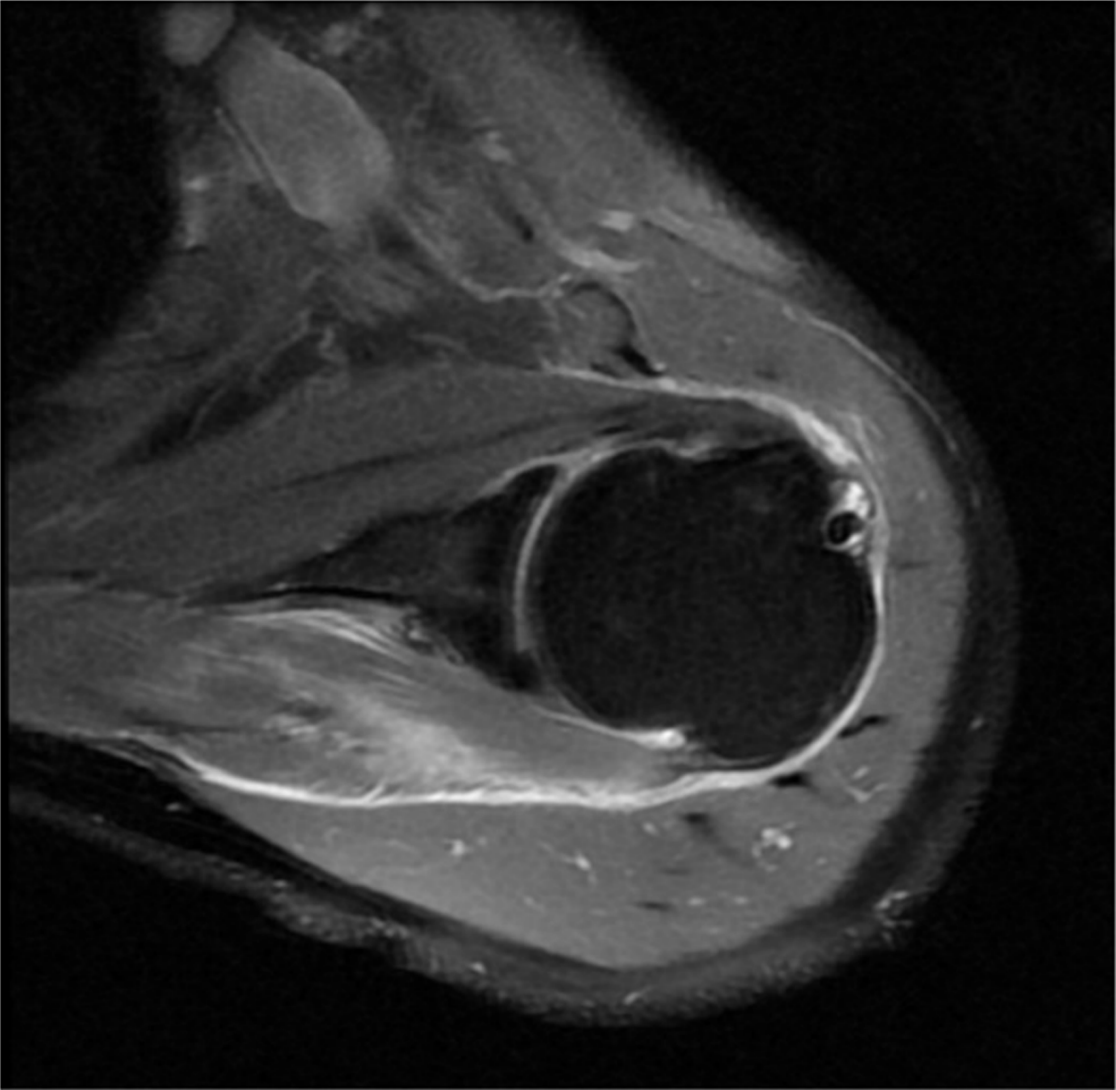
Sagittal MR image of a full-thickness rotator cuff tear.

All measurements were performed independently by two radiologists blinded to the study patients. The average of the measurements obtained by the two observers was calculated to minimize inter-observer error. As shown in Table 1, a high level of agreement was achieved between the two observers (see Figure 5).

**Table 1:** Interobserver reliability of imaging measurements.

Measurement	ICC (2,1)	95% CI	ICC (2,2)	95% CI
NAA	0.94	0.93–0.95	0.97	0.96–0.97

**Figure 5:**
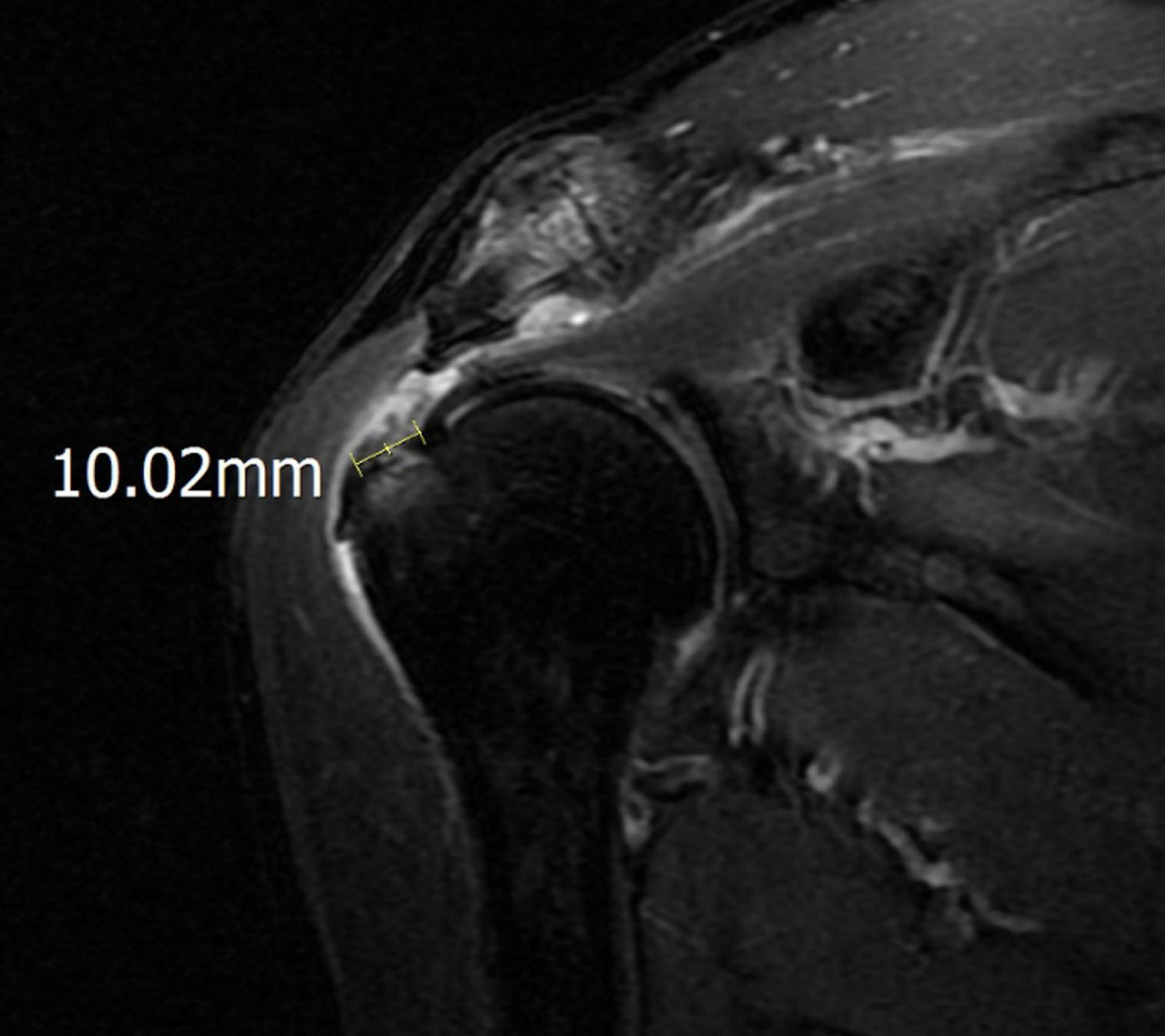
Depth of a partial bursal-side rotator cuff tear on coronal MR imaging.

### Statistical methods

Data were statistically analyzed using SPSS 25.0 software. Measurement data conforming to a normal distribution are presented as mean ± standard deviation (x̄ ± s). One-way ANOVA or its nonparametric equivalent was used for multi-group comparisons according to the normality of data distribution. Statistical significance was defined as two-tailed *α* = 0.05. The Chi-square test was used to compare the distributions of sex and affected side between groups (see Figure 6).

**Figure 6:**
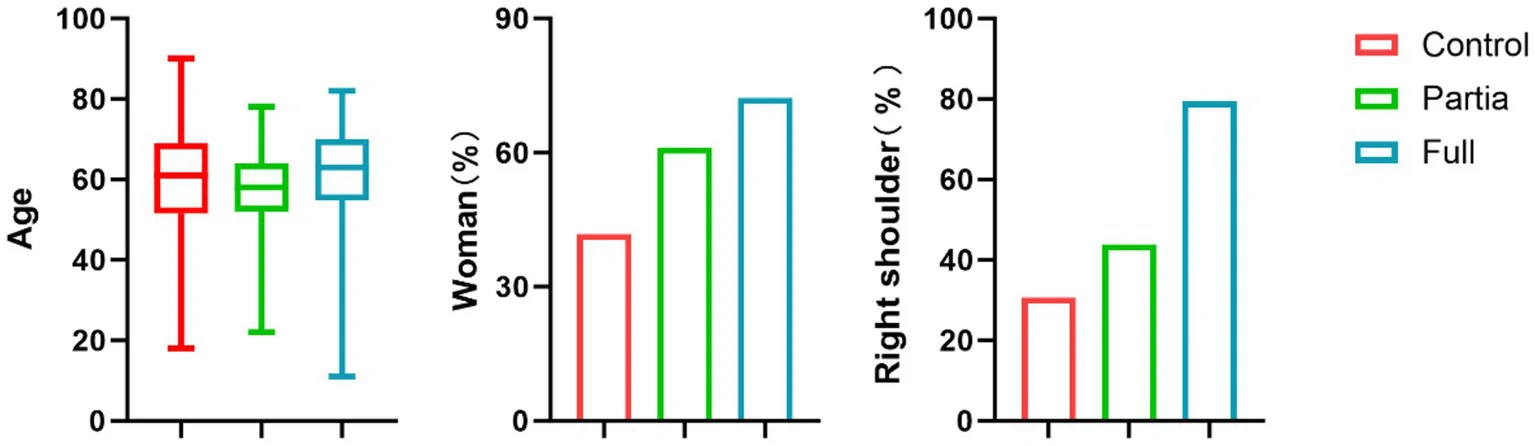
Baseline group characteristics: age, proportion of woman participants, and proportion of right shoulders.

## Results

This study included 386 patients: 134 in the symptomatic MRI-negative control group, 126 in the partial bursal-side rotator cuff tear group, and 126 in the full-thickness rotator cuff tear group. Baseline characteristics differed significantly among the three groups (Table 2; *p* < 0.01). As shown in Table 3, Figure 7, mean NAA values were 148.0 ± 7.5° in Group 1 (symptomatic MRI-negative controls), 144.0 ± 7.0° in Group 2 (partial bursal-side tears), and 141.9 ± 6.2° in Group 3 (full-thickness tears). NAA differed significantly between Group 1 and both Group 2 and Group 3 (both *p* < 0.01). Multivariable logistic regression showed that NAA was the only variable independently associated with full-thickness rotator cuff tears (adjusted OR = 0.90, 95% CI, 0.87–0.93; *p* < 0.01), whereas age, male sex, and right-sided involvement were not significantly associated with full-thickness tears (all *p* > 0.05) (Table 4), (see Figure 8). The AUC was 0.76 for Group 3, indicating better discriminatory performance than for Group 2. Smaller NAA values were also associated with larger full-thickness tear sizes. NAA did not differ significantly among the three grades of partial bursal-side tears (Table 5, Figure 9; *p* > 0.05). In the full-thickness tear group, tear size tended to increase as NAA decreased (Table 6, Figure 10).

**Table 2:** Comparison of general characteristics among the three groups.

	Control	Partial	Full	Test statistic	*p* value
Age	59.1 ± 14.5	57.7 ± 9.5	62.2 ± 10.8	*F* = 4.79	*p* < 0.01
Male/woman	78/56	49/77	35/91	*χ*^2^ = 25.42	*p* < 0.01
Left/right	93/41	70/56	26/100	*χ*^2^ = 65.06	*p* < 0.01

**Table 3:** Comparison of NAA among the three groups.

	Control	Partial	Full	*H*	*p* value	Pairwise *p* values
NAA (°)	148.0 ± 7.5	144.0 ± 7.0	141.9 ± 6.2	54.02	<0.01	Control vs. Partial <0.01
						Control vs. Full <0.01
						Partial vs. Full = 0.03

**Figure 7:**
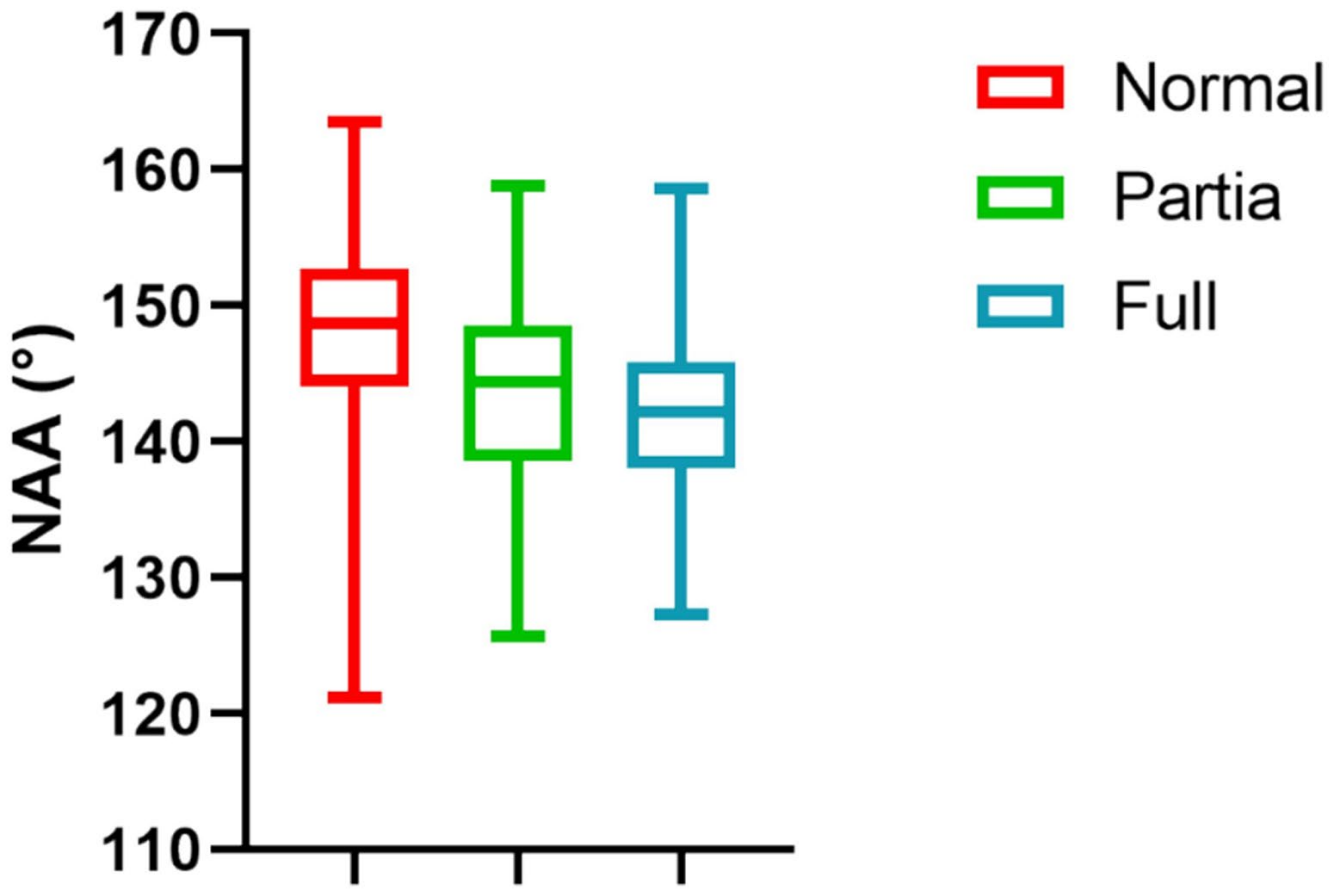
Comparison of NAA levels among the three groups.

**Table 4:** Multivariable logistic regression for full-thickness rotator cuff tear.

Predictor	Adjusted OR	95% CI	*p* value
Age	1.00	0.98–1.02	0.83
Male sex	0.80	0.51–1.26	0.34
Right shoulder	0.84	0.54–1.32	0.46
NAA	0.90	0.87–0.93	<0.01

**Figure 8:**
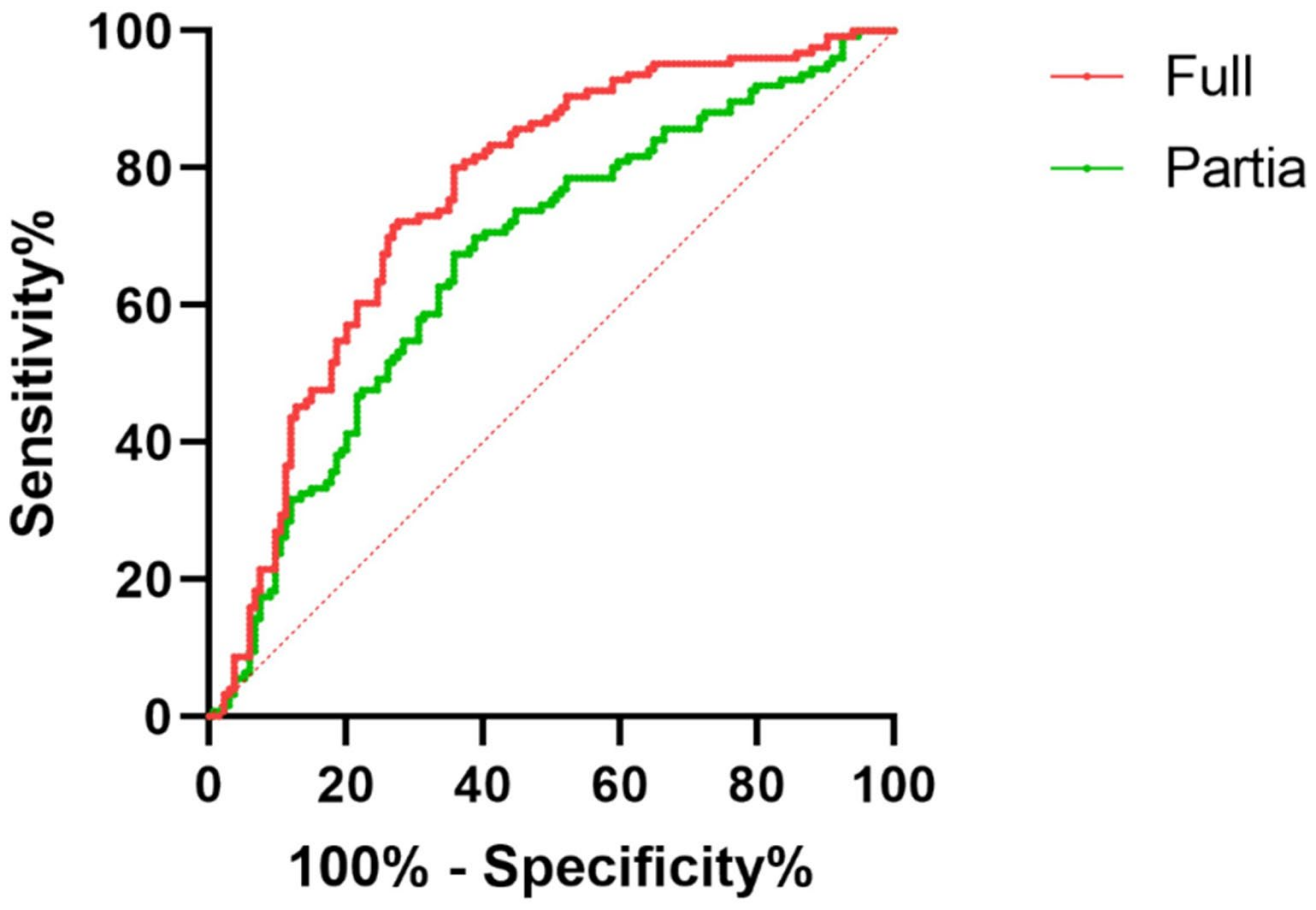
ROC curves among the three groups.

**Table 5:** Grading comparison of partial bursal-side rotator cuff tears.

	I	II	III	*F*	*p* value	Pairwise *p* values
NAA (°)	143.4 ± 6.4	143.4 ± 8.0	144.5 ± 6.7	0.33	0.72	I vs. II = 0.99
						I vs. III = 0.81
						II vs. III = 0.79

**Figure 9:**
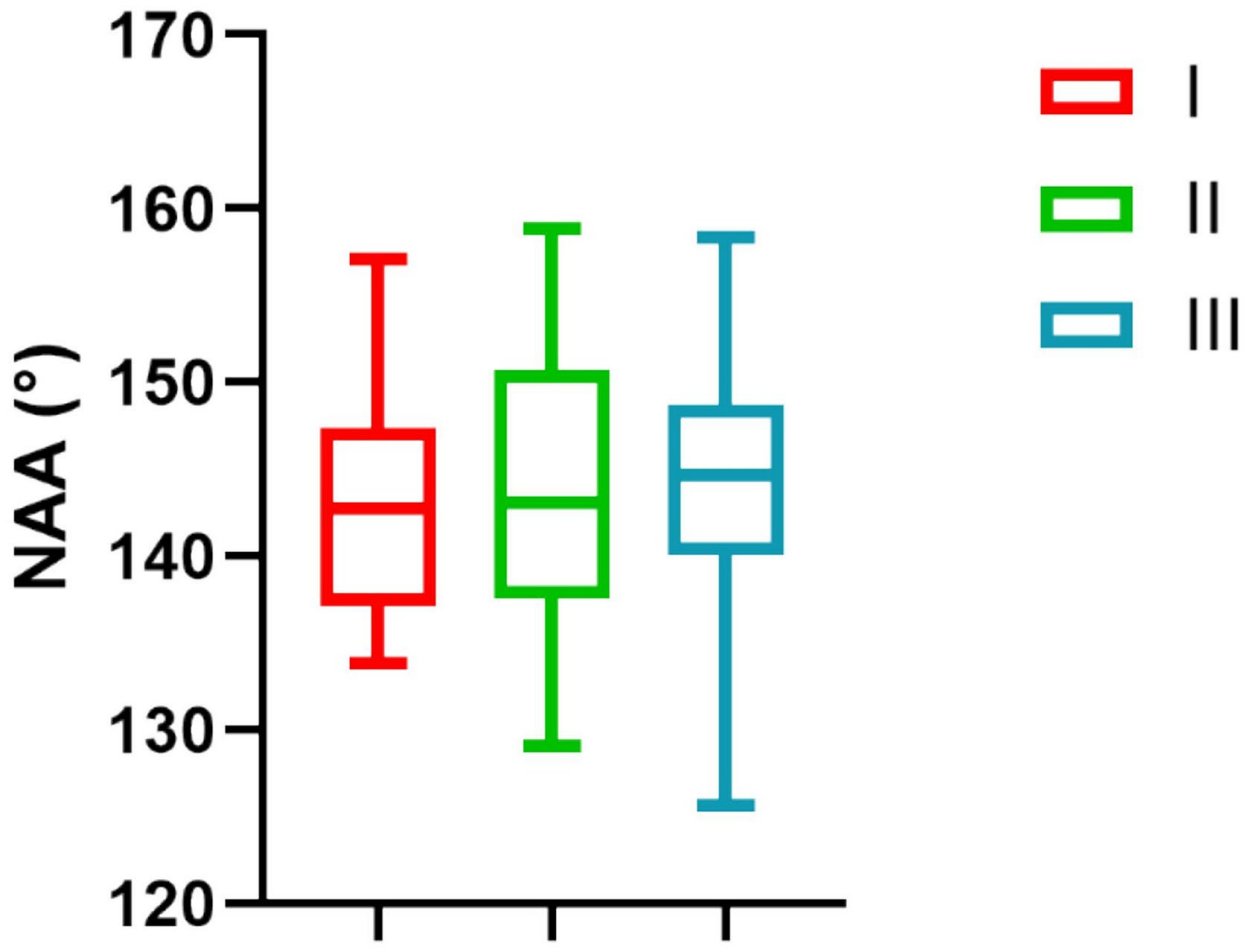
Comparison of the degree of partial bursal-side rotator cuff tears.

**Table 6:** Comparison of the Novel Acromial Angle among rotator cuff tear-size groups.

Measurement	Small^a^	Medium^b^	Large^c^	Massive^d^	*F*	*p* value
NAA (°)	142.0 ± 6.7	142.4 ± 5.8	140.0 ± 5.7	141.4 ± 10.0	1.47	0.23
Pairwise comparisons (*p* values)						
Comparison	a vs. b	a vs. c	a vs. d	b vs. c	b vs. d	c vs. d
*p* value	0.99	0.39	0.86	0.25	0.90	0.42

**Figure 10:**
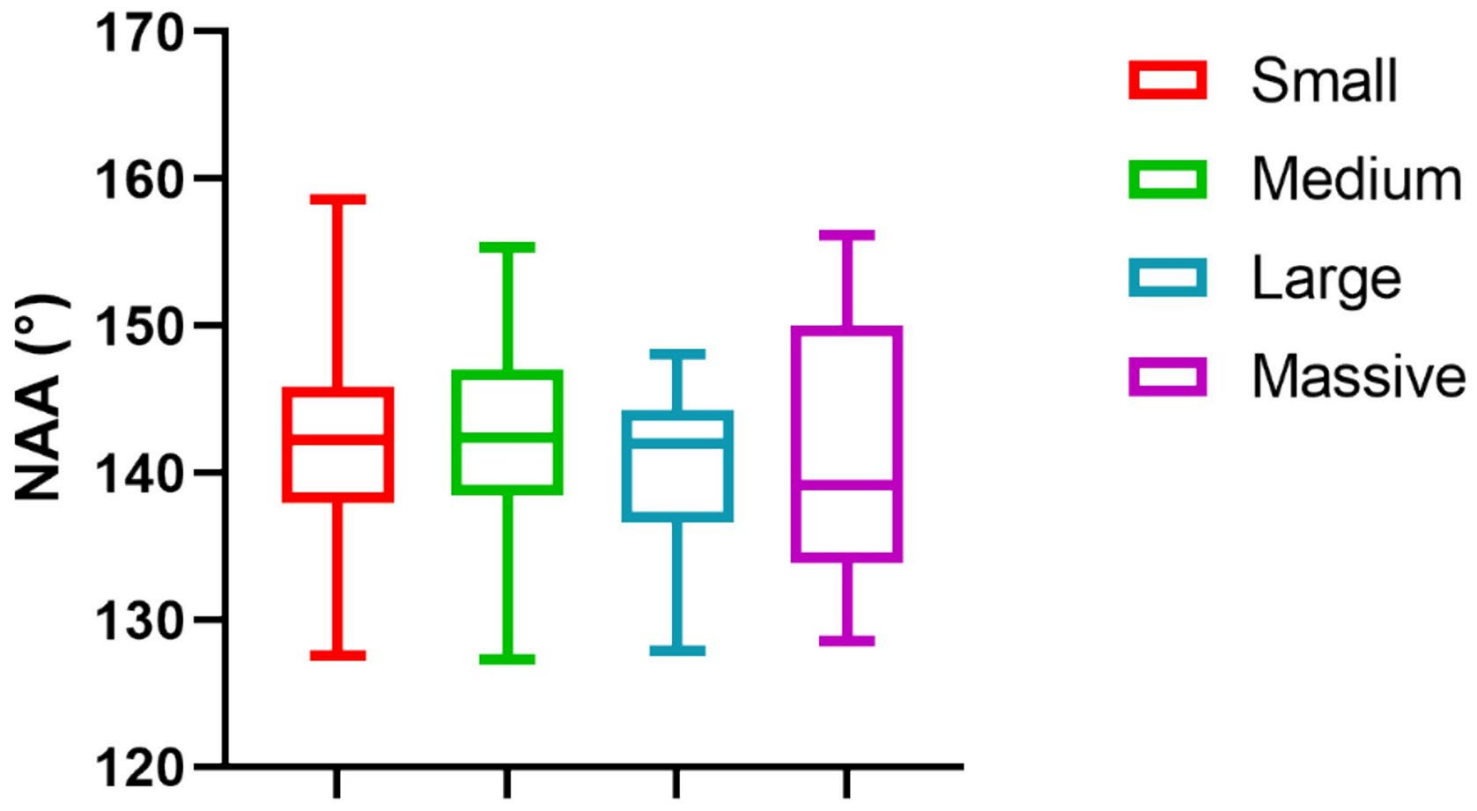
Comparison of defect sizes in the full-thickness rotator cuff tear group.

## Discussion

Numerous studies have examined the association between acromial morphology and rotator cuff tears. Moor et al. (4) introduced the critical shoulder angle (CSA) and reported that a CSA > 35° was associated with rotator cuff tears. The CSA reflects both glenoid inclination and lateral acromial extension. A larger CSA increases lateral acromial coverage and may increase glenohumeral shear forces and the load transmitted through the rotator cuff. Biomechanical analysis has further shown that supraspinatus loading increases markedly when the CSA exceeds 38°, potentially contributing to progressive tendon degeneration (8). Nyffeler et al. (19) introduced the acromion index (AI), defined as the ratio of the distance from the glenoid to the lateral acromial edge to the distance from the glenoid to the most lateral aspect of the greater tuberosity. Like the CSA, the AI reflects lateral acromial extension. In a study of 200 patients, Kum et al. (9) found that a higher AI was associated with an increased likelihood of rotator cuff tears, possibly because greater lateral extension increases supraspinatus coverage and contact pressure generated by the deltoid. In a retrospective study of 1,000 patients, Heuberer et al. (7) found that the CSA discriminated among shoulder pathologies better than the AI, with the highest CSA values in patients with rotator cuff tears and the lowest in those with osteoarthritis. Tunalı et al. (10) further evaluated the relationships of CSA and AI with tear size in 218 patients classified as symptomatic MRI-negative controls, partial bursal-side tears, or full-thickness tears. Larger CSA and AI values were associated not only with the presence of rotator cuff tears but also with larger full-thickness tears. Collectively, these findings support an association between acromial morphology and rotator cuff pathology.

Both the CSA and AI are derived from anteroposterior scapular radiographs. However, scapular outlet-view radiographs are commonly used in clinical practice to evaluate acromial morphology and the potential need for acromioplasty. The anterior third of the acromion is especially relevant because morphologic changes frequently occur in this region, which is also the primary target of acromioplasty. In 2025, Hou et al. (11) introduced the NAA and demonstrated its association with rotator cuff tears in a retrospective study of 148 patients. Building on that work, the present study evaluated the relationship between NAA and tear severity, including massive tears. Mean NAA values were 148.0 ± 7.5° in the symptomatic MRI-negative control group, 144.0 ± 7.0° in the partial bursal-side tear group, and 141.9 ± 6.2° in the full-thickness tear group. Significant differences were observed between the control group and each tear group. A smaller NAA was associated with greater partial-tear depth and larger full-thickness tear size. The NAA primarily characterizes the anterior one-third of the acromion, where morphologic changes commonly occur. Snyder et al. (12) likewise emphasized the importance of the junction between the anterior and middle thirds of the acromion and developed an acromial classification based on thickness in this region. A smaller NAA may indicate greater inferior acromial curvature and a higher likelihood of a hooked acromion. Hooked acromial morphology can increase subacromial impingement and thereby contribute to rotator cuff injury. Maman et al. (13) identified age >60 years as a risk factor for rotator cuff tear progression, possibly because age-related tendon degeneration increases tendon fragility and susceptibility to tearing.

Subacromial impingement and advancing age may promote osteophyte formation along the inferior acromial margin, thereby reducing the NAA and further exacerbating impingement in a self-reinforcing cycle. Rotator cuff tears are generally thought to arise from intratendinous degeneration characterized by extracellular matrix disorganization and inflammatory responses, with progression from partial to full-thickness tears (14). This process underscores the importance of intrinsic tendon quality in rotator cuff pathology. Yu et al. (15) reported that acromial impingement can activate coordinated mitochondrial and endoplasmic reticulum apoptotic pathways, resulting in excessive tenocyte apoptosis, tendinopathy, and increased susceptibility to tearing. Studies using three-dimensional tendon models have also shown that mechanical overload fragments mitochondrial networks and promotes the release of extracellular particles containing mitochondrial components; these particles recruit macrophages and stimulate pro-inflammatory cytokine secretion, thereby contributing to tendinopathy and rotator cuff tears. Taken together, these findings suggest that subacromial impingement and mechanical overload may aggravate tendinopathy through mitochondrial dysfunction. As a quantitative measure of acromial morphology, NAA may therefore represent an important anatomical factor associated with rotator cuff injury (16, 17).

In the present study, univariable analyses showed that age, sex, and affected side were associated with rotator cuff injury. However, multivariable logistic regression identified NAA as the only independent correlate of full-thickness rotator cuff tears. This difference likely reflects the inability of univariable analyses to account for confounding. After adjustment in the multivariable model, the associations of age, sex, and affected side were no longer statistically significant, suggesting that their crude associations may have been explained by other covariates. Advancing age is associated with both acromial osteophyte formation and degenerative narrowing of the subacromial space, which may reduce NAA. Aging also promotes rotator cuff tendon degeneration and loss of elasticity. These parallel degenerative processes may explain why age was associated with rotator cuff injury before adjustment for anatomical variables. After NAA was entered into the multivariable model, the association with age was no longer significant. Similarly, sex and affected side were not independently associated with full-thickness tears after adjustment. Nevertheless, the sample size may have limited statistical power, and larger studies are needed to validate these findings.

Full-thickness tears were classified according to Cofield (18). Relatively few large and massive tears were identified on MRI, although larger tears are frequently encountered intraoperatively. This discrepancy may reflect differences between preoperative MRI and intraoperative measurements. Previous research has demonstrated that MRI-based classification has high sensitivity and positive predictive value for medium-sized tears (100%) but lower values for larger tears (75%) (19). Thus, MRI may result in misclassification of some tear sizes, although discrepancies are less common for medium-sized tears. Eren et al. (20) found that intersurgeon agreement regarding intraoperative tear size was lower than agreement among radiologists assessing MRI. MRI nevertheless provides reasonably accurate measurements of rotator cuff tears (21, 22). To improve consistency and minimize measurement error, tear size was therefore assessed uniformly on MRI in the present study, and the continuous MRI measurements were also compared directly. For full-thickness tears, the AUC was 0.76, with a sensitivity of 72.2%, specificity of 72.4%, and Youden index of 44.6%. For partial tears, the AUC was 0.67, with a sensitivity of 64.3%, specificity of 64.2%, and Youden index of 28.5%. Thus, NAA demonstrated better discriminatory performance for full-thickness tears than for partial tears. The adjusted OR for NAA was <1, indicating that lower NAA values were associated with a greater likelihood of rotator cuff injury. The optimal NAA cutoff was 144.9°; values below this threshold were associated with a higher likelihood of rotator cuff injury, particularly full-thickness tears. Overall, NAA showed a stronger association with full-thickness tears than with partial-thickness tears. One possible explanation is that partial bursal-side tears caused by subacromial impingement may be detected and treated before progressing to full-thickness tears, limiting further changes in acromial morphology. A smaller NAA may increase the likelihood of subacromial impingement and, over time, contribute to more severe full-thickness tears. Some patients may delay assessment or treatment until the tear has enlarged or retracted. During this interval, aging (23), repeated impingement, and osteophyte formation may further decrease NAA.

Several limitations should be acknowledged. First, routine MRI was not acquired with a slice thickness of 1 mm; therefore, the true tear margins may have fallen between adjacent slices, limiting measurement precision. Second, tears were confirmed arthroscopically, whereas controls were classified using MRI alone. Because arthroscopy is not routinely performed in patients with negative imaging findings without a specific clinical indication, this difference in verification was unavoidable but may have introduced classification bias. Third, the sample size was limited, particularly in some tear-size subgroups, and larger cohorts are needed. Finally, acromial morphology may vary by ethnicity (24–26). The AI has been reported as a risk factor for rotator cuff tears in Brazilian populations but not in Japanese populations (27). Ethnic variation was not evaluated in the present study and should be addressed in future multicenter studies.

## Data Availability

The original contributions presented in the study are included in the article/supplementary material, further inquiries can be directed to the corresponding authors.
